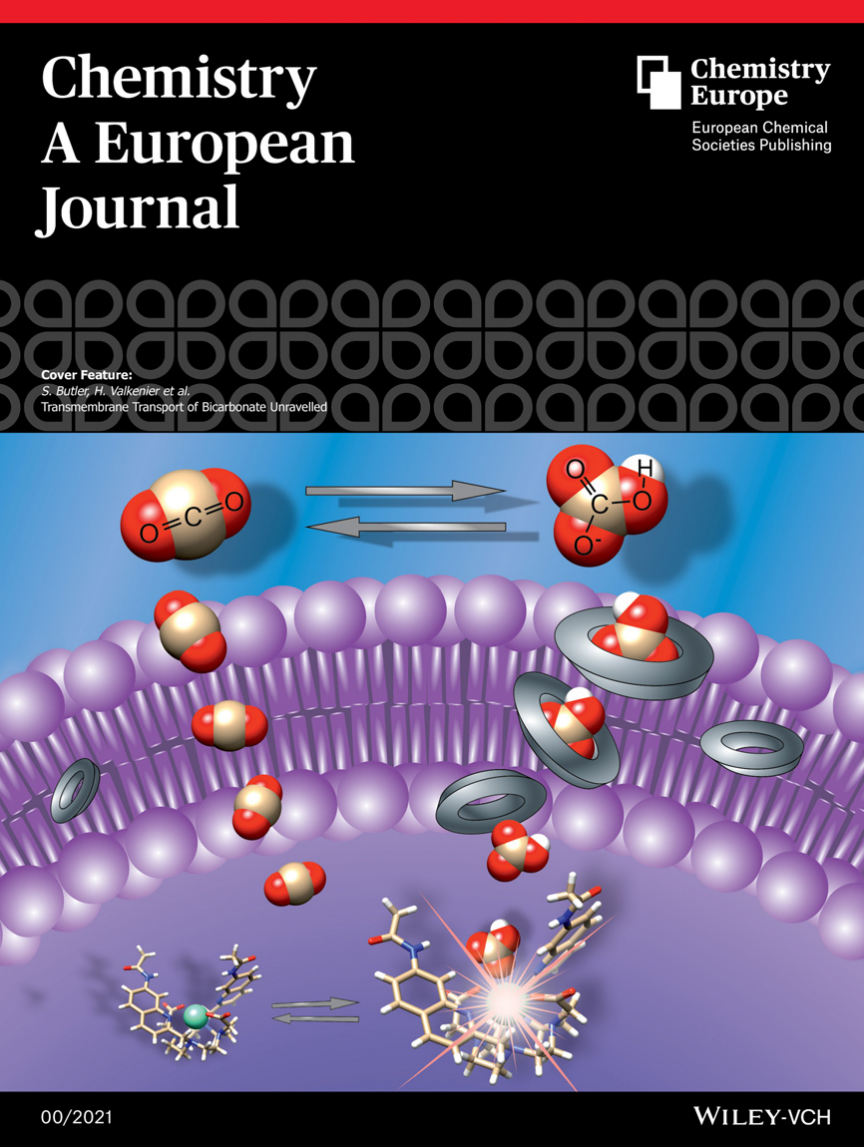# Transmembrane Transport of Bicarbonate Unravelled

**DOI:** 10.1002/chem.202101345

**Published:** 2021-05-06

**Authors:** Luis Martínez‐Crespo, Sarah H. Hewitt, Nicola Alessandro De Simone, Vladimír Šindelář, Anthony P. Davis, Stephen Butler, Hennie Valkenier

**Affiliations:** ^1^ Université Libre de Bruxelles (ULB) Engineering of Molecular NanoSystems, Ecole polytechnique de Bruxelles Avenue F.D. Roosevelt 50, CP165/64 1050 Brussels Belgium; ^2^ Loughborough University Department of Chemistry Epinal Way Loughborough LE11 3TU UK; ^3^ Masaryk University Department of Chemistry and RECETOX, Faculty of Science Kamenice 5 625 00 Brno Czech Republic; ^4^ University of Bristol School of Chemistry Cantock's Close Bristol BS8 1TS UK

## Abstract

**Invited for the cover of this issue are Dr. Stephen Butler, Dr. Hennie Valkenier and co‐workers at Université Libre de Bruxelles, Loughborough University, Masaryk University, and the University of Bristol. The image depicts the transport of bicarbonate anions versus the spontaneous diffusion of CO_2_ across the lipid bilayer of a liposome. Read the full text of the article at** 10.1002/chem.202100491.

## Graphical abstract

“*The biggest surprise in this research was the observation of apparent bicarbonate transport by cation transporters. Further investigations using our new assay showed that this could be attributed to a combination of proton transport and CO_2_ diffusion*.” Read more about the story behind the cover in the Cover Profile and about the research itself on page 7319 ff. (DOI: 10.1002/chem.202100491).

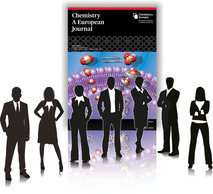

## What was the inspiration for this cover design?


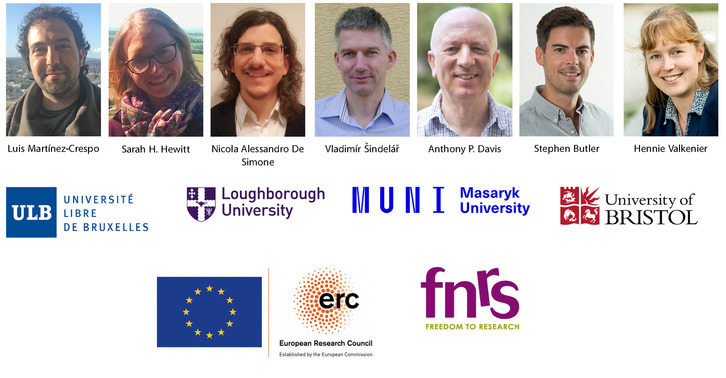


The cover image was designed by Luis Martínez‐Crespo and depicts the transport of bicarbonate anions versus the spontaneous diffusion of CO_2_ across the lipid bilayer of a liposome. In our contribution, we show how these processes can be monitored by a single europium(III) complex, of which the emission intensity increases in the presence of bicarbonate.

## What prompted you to investigate this topic?

This contribution originates from the overarching aim of Hennie Valkenier to develop transporters for various biologically relevant ions, including bicarbonate and phosphates. Fluorescence spectroscopy on liposomes with an encapsulated probe is a convenient way to study such transport processes, but this requires either the employment of indirect methods (monitoring the transport of other ions than the one of interest) or the development of new methodology, which is what we present in this article.

## How did the collaboration on this project start?

Hennie Valkenier and Stephen Butler met at a conference and realised that the europium complexes developed by Stephen could find applications as probes in transport studies. Previous work with Tony Davis and Vladimír Šindelář had already resulted in the identification of promising bicarbonate transporters, and it was thus a logical choice to study these compounds in more detail with our new methodology.

## What was the biggest surprise?

The biggest surprise in this research was the observation of apparent bicarbonate transport by cation transporters. Further investigations using our new assay showed that this could be attributed to a combination of proton transport and CO_2_ diffusion. We expect that the ability to distinguish between the mechanisms of bicarbonate uptake will facilitate the design of more selective and efficient bicarbonate transporters in the future.